# Spacecraft Homography Pose Estimation with Single-Stage Deep Convolutional Neural Network

**DOI:** 10.3390/s24061828

**Published:** 2024-03-12

**Authors:** Shengpeng Chen, Wenyi Yang, Wei Wang, Jianting Mai, Jian Liang, Xiaohu Zhang

**Affiliations:** School of Aeronautics and Astronautics, Sun Yat-sen University, Shenzhen 510275, China; chenshp8@mail2.sysu.edu.cn (S.C.); yangwy58@mail2.sysu.edu.cn (W.Y.); wangw376@mail2.sysu.edu.cn (W.W.); maijt8@mail2.sysu.edu.cn (J.M.); liangj235@mail2.sysu.edu.cn (J.L.)

**Keywords:** spacecraft, pose estimation, homography, single-stage, geometric constraint, 2D keypoints

## Abstract

Spacecraft pose estimation using computer vision has garnered increasing attention in research areas such as automation system theory, control theory, sensors and instruments, robot technology, and automation software. Confronted with the extreme environment of space, existing spacecraft pose estimation methods are predominantly multi-stage networks with complex operations. In this study, we propose an approach for spacecraft homography pose estimation with a single-stage deep convolutional neural network for the first time. We formulated a homomorphic geometric constraint equation for spacecraft with planar features. Additionally, we employed a single-stage 2D keypoint regression network to obtain homography 2D keypoint coordinates for spacecraft. After decomposition to obtain the rough spacecraft pose based on the homography matrix constructed according to the geometric constraint equation, a loss function based on pixel errors was employed to refine the spacecraft pose. We conducted extensive experiments using widely used spacecraft pose estimation datasets and compared our method with state-of-the-art techniques in the field to demonstrate its effectiveness.

## 1. Introduction

The lighting environment in space differs from that on the ground, as light propagates linearly, resulting in intense light reflection on the surface of spatial targets. In contrast, there are non-reflective parts on the surface of spatial targets, leading to missing parts of the target during the imaging process [1]. On the other hand, the high-speed motion of orbiting spacecraft introduces motion blur during the imaging process [2]. Therefore, images of spacecraft in space exhibit characteristics such as overexposure, underexposure, occlusion, partial target loss, and high contrast. For template matching methods that rely on target texture, shape, and contours, significant challenges are encountered. Under such conditions, constructing a dense and accurate spacecraft model is highly challenging, and it incurs a significant computational cost [3,4].

Computer vision-based spacecraft pose estimation is increasingly garnering attention from research domains such as automation system theory, control theory, sensors and instruments, production systems, manufacturing engineering, robotics technology, and automation software. The determination of spacecraft pose is a prerequisite and foundation for spacecraft control. Traditional target pose estimation is primarily based on image preprocessing operations. Specifically, target pose is determined by solving the projection equation through establishing correspondences between 2D and 3D or between 3D and 3D keypoints. Examples of such feature extraction methods include SIFT [5], FAST [6], SURF [7], ORB [8], FPFH [9], etc. However, in some challenging scenarios with extreme imaging conditions, these methods suffer from drawbacks such as high computational complexity, error-prone matching, and low accuracy. With the continuous development of deep learning, spacecraft pose estimation with convolutional neural networks is increasingly gaining attention in on-orbit research. Sharma et al. introduced a spacecraft pose network (SPN) for the monocular visual pose estimation of known non-cooperative spacecraft [10,11,12]. We propose an approach for spacecraft homography pose estimation with a single-stage deep convolutional neural network. Firstly, compared to the common two-stage SPN, this method achieves simultaneous spacecraft detection and feature extraction tasks based on the single-stage neural network of SPN. Secondly, the method can accurately estimate the pose of spacecraft based on the coplanar features of the spacecraft. Thirdly, in challenging scenarios and domain gap situations, our proposed research method remains robust and effective.

Currently, research on spacecraft pose estimation based on neural networks has emerged as a new area of focus in computer vision. Bo Chen et al. employed a two-stage deep learning approach incorporating prior reconstruction of a 3D model [13], achieving state-of-the-art performance in the 2019 Satellite Pose Estimation Challenge (SPEC2019) [14]. In consequence of the unique nature of the space environment, acquiring real images with rich labels is highly constrained. Therefore, Unreal Engine 4 is utilized to render the on-orbit flights of known non-cooperative spacecraft, thereby obtaining widely used simulated datasets [15,16]. In 2021, the Advanced Concepts Team (ACT) of the European Space Agency and the Space Rendezvous Laboratory (SLAB) of Stanford University organized the Satellite Pose Estimation Challenge (SPEC2021) [17]. Differing from SPEC2019, SPEC2021 placed special emphasis on domain gaps.

## 2. Related Work

### 2.1. Multi-Stage Pose Estimation

Multi-stage pose estimation refers to the utilization of various strategies to accomplish different tasks. These tasks are executed in a specified sequence to obtain the final pose estimation. A multi-stage network can be composed of multiple sub-networks, for example, employing three sub-networks to address pose estimation and target detection problems. Furthermore, these three sub-networks correspondingly address orientation classification, position classification, and target detection [18]. Alternatively, two sub-networks are employed to address pose estimation, where these two sub-networks correspond to a keypoint regression network and a pose estimation network, respectively [19]. Lei Zhou et al. proposed a two-stage deformation-and-registration pipeline named DR-Pose. In the first stage of the network, occluded target point cloud restoration is accomplished, while in the second stage the target pose is estimated through voting with the completed point cloud serving as a prior reference [20]. Park et al. employed a two-stage method for non-cooperative spacecraft pose estimation. The network first utilizes a detection network to crop the region of interest around the target, and then employs a classification network for feature extraction within the region of interest [21].

### 2.2. Single-Stage Pose Estimation

Differing from multi-stage pose estimation, single-stage pose estimation is comparatively simpler in terms of network architecture. A single-stage pose estimation network comprises only one network architecture without multiple sub-networks. Additionally, a single-stage network can accomplish specified tasks or the simultaneous execution of multiple tasks. Yinlin Hu et al. introduced a grouping feature aggregation scheme that effectively addresses the corresponding clusters in object pose estimation [22]. Penglei Liu et al. introduced a multi-directional feature pyramid network that can effectively tackle occlusion and texture-less issues [23]. Tekin et al. proposed a fast and accurate monocular pose estimation neural network framework that extends single-view 2D object detection to the paradigm of 6D object detection [24].

### 2.3. Homography Pose Estimation

Homography pose estimation involves utilizing a reversible mapping with straight-line preservation between two 2D projection planes for target pose estimation. In the Perspective-n-Point problem, a minimum of three points is sufficient to recover the pose between the target and the camera. These geometric constraints can be referred to as Perspective-Three-Point. The geometric constraint equations of Perspective-Three-Point can be decomposed to obtain the pose using the Wu–Ritt zero decomposition algorithm [25]. Shiqi Li et al. proposed a research approach for homography pose estimation using Perspective Similar Triangle, transforming the Perspective-Three-Point constraint equations into quartic equations [26,27]. Additionally, when there are constraints in the spatial mapping where four points are coplanar but not collinear, the geometric constraint equations can be described as the projection relationship between quadrilaterals on two planes [28,29]. In this scenario, the pose between the target and the camera can be decomposed using the quadrilateral pose estimation algorithm [30].

## 3. Approach

**Figure 1:**
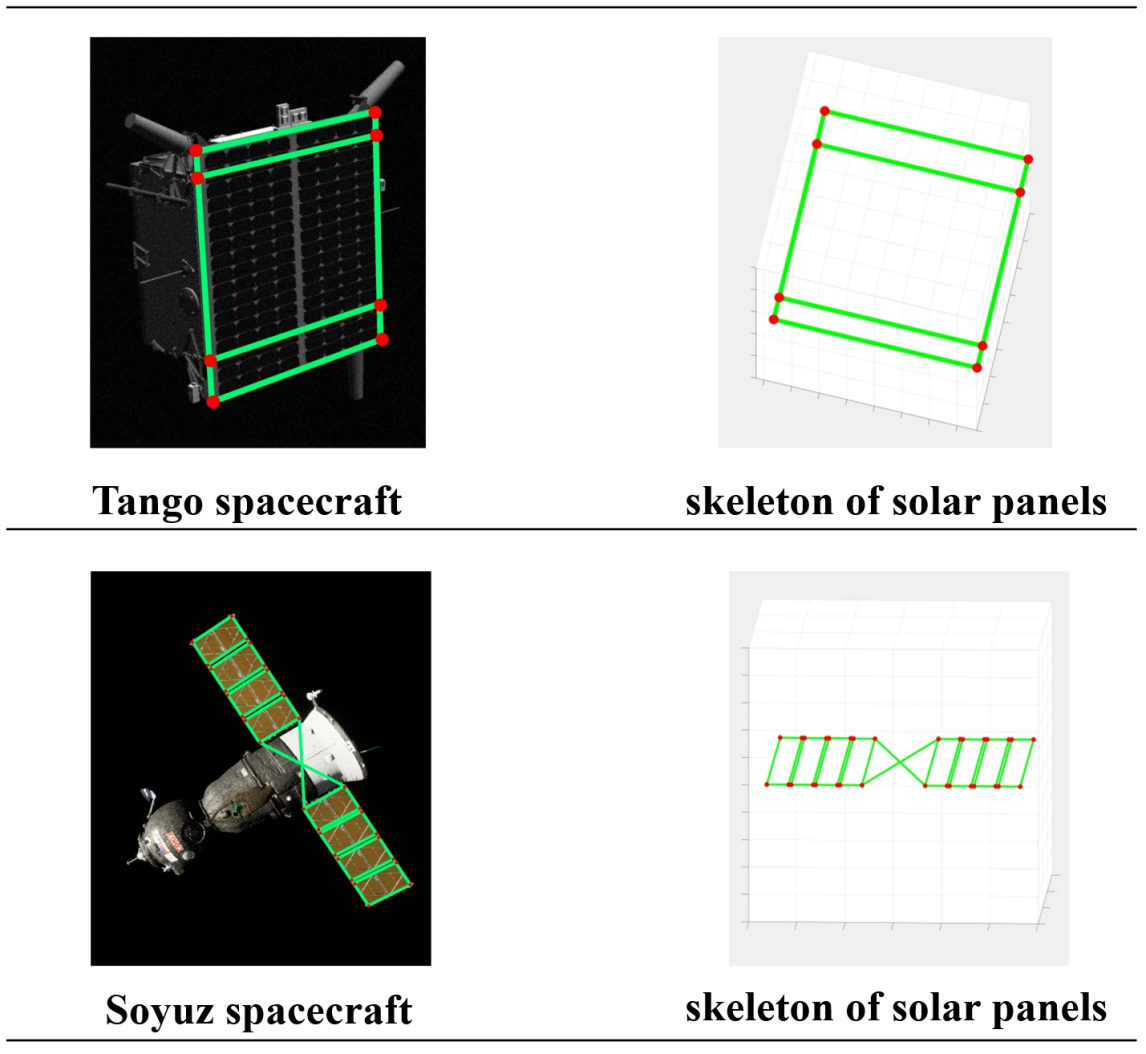
The overall structure of the spacecraft on the image and the skeleton of the spacecraft’s solar panels.

The framework under investigation in this paper is primarily composed of three main components. Firstly, we employ multi-view triangulation to construct a 3D skeleton model of spacecraft coplanar components based on a dataset with ground-truth poses, as illustrated in Figure 1. We utilize the 3D skeleton model and ground-truth poses to create the necessary training labels, denoted as I. These labels I encompass the bounding box information of coplanar spacecraft components (such as solar panels) and the 2D keypoint information of these components, as illustrated in Equation (3). Secondly, we input the training dataset into a single-stage spacecraft pose estimation network for training to obtain a weighted model capable of detecting coplanar spacecraft components and their 2D keypoints in images. The training dataset is sourced from the SPEC2021 Dataset and the URSO Soyuz Dataset, which will be described in detail in Section 4.1. The training dataset describes various poses of the Tango spacecraft and the Soyuz spacecraft in space. Additionally, the training dataset also includes labels I for both spacecraft. The test images are then fed into the single-stage spacecraft pose estimation network, resulting in the extraction of the regions of interest in the images and keypoint information for the coplanar components of the spacecraft. Finally, we input the obtained 2D keypoint information of the spacecraft and the 3D model of the spacecraft into the pose estimation solver. The initial rough spacecraft pose is acquired through a homography pose estimation algorithm and subsequently refined through nonlinear optimization to obtain the refined pose.

### 3.1. 3D Reconstruction and Reprojection

**Figure 2:**
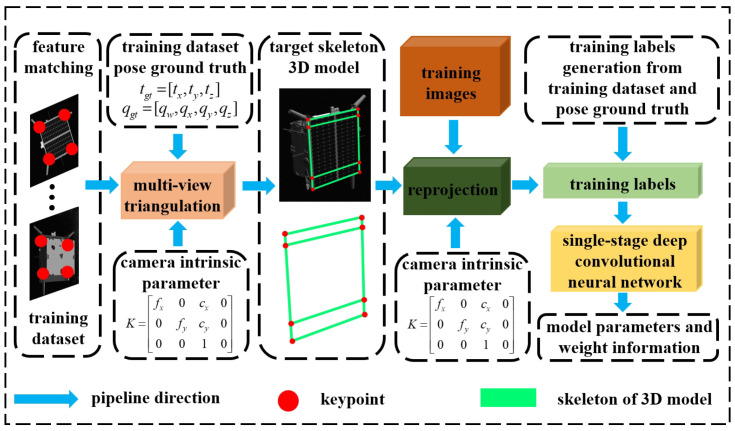
Illustrating the pipeline for the reconstruction process of the spacecraft 3D skeleton model and the generation of training labels.

In accordance with the images and ground-truth pose information within the training dataset, we employ a multi-view triangulation approach to construct the 3D skeleton model of the spacecraft, as depicted in Equation (1). The projection matrix M is defined as $M={\left[M_{0},M_{1},M_{2}\right]}^{T}$, where $M_{0}$, $M_{1}$, and $M_{2}$ represent the row vectors of M. Here, K represents the camera intrinsic parameters, and $R_{gt}$ and $T_{gt}$ denote the rotation matrix and translation vector of the pose, respectively, with gt indicating ground truth. The notation e(u,v) denotes the 2D keypoint of the spacecraft, where *u* and *v* represent the two components of the image coordinates e in the vertical and horizontal directions, respectively. P(X,Y,Z) represents the coordinates of the spacecraft 3D keypoint. Here, *z* denotes the depth of the spacecraft keypoint perpendicular to the image plane in the camera coordinate system, while *i* represents the image id and *j* represents the keypoint id. We reformulate Equation (1) into a system of linear equations, as depicted in Equation (2). The 3D information of spacecraft keypoint is reconstructed by solving Equation (2), as illustrated in Figure 2. We utilize the ground-truth poses from the training dataset to reproject the 3D skeleton model of the spacecraft onto images, following Equation (1). This process yields the 2D coordinates of the spacecraft 3D skeleton model keypoints in the images. Subsequently, we use this information to construct the labels, denoted as I, required for training, as depicted in Equation (3). In the given expression, $c_{id}$ denotes the target classification, which can be represented by natural numbers such as 0, 1, 2, and so forth, while ($c_{x},c_{y},w,h$) respectively denote the center coordinates and dimensions of the target region of interest, with the • serving as the normalization indicator. Assuming the reprojected 2D keypoint set is denoted by {e(u,v)}, we can obtain $x_{min}$, $x_{max}$, $y_{min}$, and $y_{max}$ by calculating $x_{min}=min\left\{u_{j}\right\}$, $x_{max}=max\left\{u_{j}\right\}$, $y_{min}=min\left\{v_{j}\right\}$, and $y_{max}=max\left\{v_{j}\right\}$. Based on these four values, we can obtain $\dot{c_{x}}=\frac{\left(x_{max}\;+\;x_{min}\right)}{2w}$, $\dot{c_{y}}=\frac{\left(y_{max}\;+\;y_{min}\right)}{2h}$, $\dot{w}=\frac{\left(x_{max}\;-\;x_{min}\right)}{w}$, $\dot{h}=\frac{\left(y_{max}\;-\;y_{min}\right)}{h}$, $\dot{u_{j}}=\frac{u_{j}}{w}$, and $\dot{v_{j}}\frac{v_{j}}{h}$, where *w* and *h* represent the width and height of the image, respectively.

(1) $$\begin{array}{c} \left\{\begin{array}{c} z_{j}^{\left(i\right)}\left[\begin{array}{c} e_{j}^{\left(i\right)}\\ 1 \end{array}\right]=K{\left[\begin{array}{cc} R_{gt} & T_{gt}\\ 0 & 1 \end{array}\right]}^{\left(i\right)}\left[\begin{array}{c} P_{j}\\ 1 \end{array}\right]\\ M^{\left(i\right)}=K{\left[\begin{array}{cc} R_{gt} & T_{gt}\\ 0 & 1 \end{array}\right]}^{\left(i\right)} \end{array}\right. \end{array}$$

(2) $$\begin{array}{c} \left\{\begin{array}{c} P_{j}^{T}M_{0}^{(i)T}-u_{j}^{\left(i\right)}P_{j}^{T}M_{2}^{(i)T}=0\\ P_{j}^{T}M_{1}^{(i)T}-v_{j}^{\left(i\right)}P_{j}^{T}M_{2}^{(i)T}=0 \end{array}\right. \end{array}$$

(3) $$\begin{array}{c} I_{i}=\left\{c_{id},\;\dot{c_{x}},\;\dot{c_{y}},\;\dot{w},\;\dot{h},\;\dot{u_{1}},\;\dot{v_{1}},\;\ldots ,\;\dot{u_{j}},\;\dot{v_{j}}\;\ldots \right\} \end{array}$$

### 3.2. Single-Stage Spacecraft Pose Network

#### 3.2.1. Bounding Box Loss Function

The detector for the single-stage spacecraft keypoint regression network is based on Complete Intersection over Union [31] (CIoU). The CIoU loss exhibits scale invariance and can directly optimize evaluation metrics. The bounding box (bbox) loss function is depicted in Equation (4). The single-stage spacecraft keypoint regression network can predict multiple anchors, where *k*, (x,y), and *s*, respectively, denote the anchor id, position, and scale. gt stands for the identifier of the ground truth, and pred represents the prediction.

(4) $$\begin{array}{c} L_{b}\left(s,x,y,k\right)=1-R_{CIoU}\left(bbox_{gt},bbox_{pred}\right) \end{array}$$

#### 3.2.2. Keypoint Regression Loss Function

**Figure 3:**
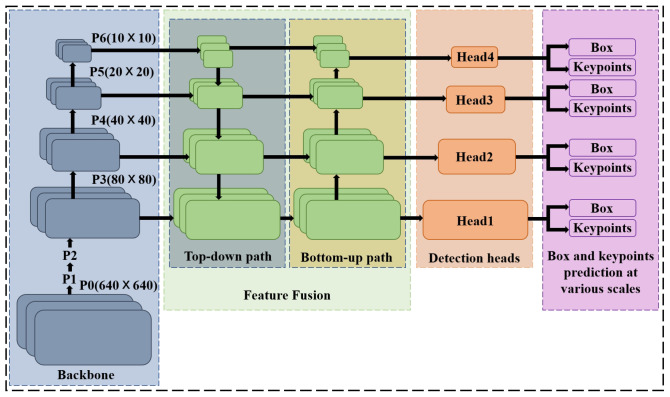
Illustrating the architecture of the single-stage 2D keypoint regression network [32]. The input image is passed through the backbone [35] that generates feature maps at various scales: P3, P4, P5, P6. The output of feature fusion [36] is fed to the detection heads. Each detection head branches into a box head and keypoint head.

The single-stage spacecraft keypoint regression network directly regresses keypoints based on the center of the anchor [32,33]. Object Keypoint Similarity [34] (OKS) is considered as Intersection over Union (IoU), and the OKS loss exhibits scale invariance. The bbox stores information about a keypoint. Therefore, if the bbox matches an anchor located at position (x,y) with a scale of *s*, the single-stage spacecraft keypoint regression network predicts the keypoints relative to the center of the anchor. For each keypoint, OKS is calculated individually, then they are summed to obtain the final OKS loss, as depicted in Equation (5). In the provided expression, *d* represents the Euclidean distance between the predicted and ground-truth locations for the keypoint, kw denotes keypoint-specific weights, *s* represents the scale of an object, δ is the visibility flag for each keypoint, and $N_{kpts}$ represents the number of keypoints on the spacecraft. Each keypoint corresponds to a confidence parameter. The visibility flag of keypoints is used as the ground truth. Binary Cross-Entropy (BCE) loss is employed to measure the difference between the model output and the ground-truth labels. Therefore, the keypoint confidence loss function is as depicted in Equation (6), where *c* represents the predicted confidence for the keypoint. When there are multiple classes for the spacecraft, Cross-Entropy (CE) loss is employed to measure the accuracy of the model predictions for target categories, as depicted in Equation (7). Here, $N_{bbox}$ and $N_{cls}$ represent the number of bounding boxes and the number of categories, respectively. *y* and $\hat{y}$ denote the labels and predicted probabilities for the categories, respectively. In summary, we can derive the total loss for the single-stage spacecraft keypoint regression network, as depicted in Equation (8). $\lambda_{c}$, $\lambda_{b}$, $\lambda_{o}$, and $\lambda_{p}$ are hyperparameters for $L_{c}$, $L_{b}$, $L_{o}$, and $L_{p}$, respectively, and these hyperparameters can be selected based on different scales. Figure 3 illustrates the overall architecture of the keypoint regression network.

(5) $$\begin{array}{c} \left\{\begin{array}{c} L_{o}=1-\sum_{n=1}^{N_{kpts}}OKS\\ \sum_{n=1}^{N_{kpts}}OKS=\frac{\sum_{n=1}^{N_{kpts}}exp\left[\frac{1}{2}{\left(\frac{d^{\left(n\right)}}{s^{\left(n\right)}kw^{\left(n\right)}}\right)}^{2}\right]\delta }{\sum_{n=1}^{N_{kpts}}\delta } \end{array}\right. \end{array}$$

(6) $$\begin{array}{c} L_{p}=\sum_{n=1}^{N_{kpts}}BCE\left(\delta ,c^{\left(n\right)}\right) \end{array}$$

(7) $$\begin{array}{c} L_{c}=-\frac{1}{N_{bbox}}\sum_{i=1}^{N_{bbox}}\sum_{j=1}^{N_{cls}}y^{\left(i,j\right)}log\left({\hat{y}}^{\left(i,j\right)}\right) \end{array}$$

(8) $$\begin{array}{c} L_{t}=\sum_{s,x,y,k}\left(\lambda_{c}L_{c}+\lambda_{b}L_{b}+\lambda_{o}L_{o}+\lambda_{p}L_{p}\right) \end{array}$$

### 3.3. Pose Initialization and Refinement

Based on the 3D skeleton model of the spacecraft and keypoint information output by the network, we can establish geometric constraint equations. By solving this equation, the rough pose of spacecraft can be obtained. The rough pose serves as the initial input to a nonlinear optimizer, ultimately yielding the refined pose. Spacecraft exhibit distinct coplanar features, as observed in spacecraft like Tango. Alternatively, it may be that a spacecraft has only coplanar feature information that can be utilized, while other characteristic information is challenging to exploit, as in the case of the Soyuz spacecraft.

#### 3.3.1. Pose Initialization

We utilize components such as the spacecraft’s solar panels, antennas, or other coplanar features to construct a homographic geometric constraint equation. The mapping relationship between 3D spacecraft keypoints and 2D image keypoints is described by a homography matrix H. The 3D keypoint P must satisfy Equation (9), where n is the normal vector of the plane and *d* is the offset term of the plane equation. The homography matrix H can be obtained through Equation (10), where *p* represents the 2D keypoint and *I* and *S* denote the identifiers for the image plane and coplanar components of the spacecraft, respectively. The homography matrix H has 8 degrees of freedom, hence requiring only 4 keypoints to linearly recover its vector form. The rotation matrix R and translation vector T can be obtained by decomposing H through the quadrilateral pose estimation algorithm [30].

(9) $$\begin{array}{c} n^{T}P+d=0 \end{array}$$

(10) $$\begin{array}{c} \left\{\begin{array}{c} p_{I}=K_{3\times 3}\left(R-\frac{Tn^{T}}{d}\right)K_{3\times 3}^{-1}\;p_{S}\\ H=K_{3\times 3}\left(R-\frac{Tn^{T}}{d}\right)K_{3\times 3}^{-1}\;\\ P=K_{3\times 3}^{-1}\;p_{S} \end{array}\right. \end{array}$$

#### 3.3.2. Pose Refinement

The rotation matrix R and translation vector T obtained by decomposing H are considered to be the rough pose of the spacecraft. The rough pose can only be refined through nonlinear optimization. The pixel coordinates of the 3D keypoint P on the image are denoted as p, and the reprojected pixel coordinates are denoted as $p^{\prime }$. Therefore, we construct the loss function for nonlinear pose optimization based on pixel errors, as depicted in Equation (11).

(11) $$\begin{array}{c} L_{pose}=\sum_{n=1}^{N_{kpts}}{∥p-p^{\prime }∥}^{2} \end{array}$$

### 3.4. Evaluation Metrics for Pose Estimation

The evaluation metric for pose estimation accuracy is calculated in two steps [17]. We first calculate the position score $S_{t}^{\left(i\right)}$ and orientation score $S_{q}^{\left(i\right)}$, and then add these two results together, as depicted in Equation (12). gt denotes ground truth, est denotes estimated value, and q represents the quaternion form of the rotation matrix. The final pose score is obtained by calculating the average. In SPEC2021, considering hardware device precision, $S^{\left(i\right)}=0$ when $S_{t}^{\left(i\right)}<0.002173$ and $S_{q}^{\left(i\right)}<0.169^{\circ }$. The unit of evaluation for spacecraft orientation estimation is expressed in radians (rad) or degrees (°) [13,15,17].

(12) $$\begin{array}{c} \left\{\begin{array}{c} S^{\left(i\right)}=S_{q}^{\left(i\right)}+S_{t}^{\left(i\right)}\\ S_{q}^{\left(i\right)}=2arccos\left(\left|\langle q_{est}^{\left(i\right)},q_{gt}^{\left(i\right)}\rangle \right|\right)\\ \Delta t^{\left(i\right)}={\left|T_{gt}^{\left(i\right)}-T_{est}^{\left(i\right)}\right|}_{2}\\ S_{t}^{\left(i\right)}=\frac{\Delta t^{\left(i\right)}}{{\left|T_{gt}^{\left(i\right)}\right|}_{2}} \end{array}\right. \end{array}$$

Additionally, the Average Distance of Descriptors (ADD) metric is applied to calculate the average distance between corresponding points of 3D models with the estimated pose and ground-truth pose [24]. If the calculated $\omega_{ADD}$ is less than 0.1 times the equivalent diameter of the target model, it is considered accurate, and the computed pose is deemed valid. Finally, the accuracy of the pose estimation method is demonstrated by statistically analyzing valid poses. Here, M denotes the set of 3D keypoints for the target skeleton, and P represents the 3D keypoint of the target, as depicted in Equation (13).

(13) $$\begin{array}{c} \omega_{ADD}=\underset{P\in M}{avg}∥\left(R_{gt}P+T_{gt}\right)-\left(R_{est}P+T_{est}\right)∥ \end{array}$$

## 4. Experiments

### 4.1. Dataset Analysis

#### 4.1.1. Satellite Pose Estimation Challenge 2021

The SPEC2021 dataset comprises three components: lightbox dataset, sunlamp dataset, and synthetic dataset. The lightbox dataset consists of 6740 real images captured under lightbox conditions. The sunlamp dataset consists of 2791 real images captured under sunlamp conditions. The real images faithfully reproduce the Earth albedo and direct sunlight as seen in satellite imagery. The dataset contains 9531 Hardware-In-the-Loop (HIL) test images of the half-scale mockup model of the Tango spacecraft captured from the Testbed for Rendezvous and Optical Navigation (TRON) facility at Stanford’s Space Rendezvous Laboratory (SLAB) [17]. The 60,000 synthetic images of the Tango spacecraft are created using the camera emulator software of the Optical Stimulator. The software uses the OpenGL-based image rendering pipeline to generate photo-realistic images of the Tango spacecraft with desired ground-truth poses [14]. Furthermore, the validation dataset and training dataset are partitioned in a 1:4 ratio. The lightbox dataset and sunlamp dataset serve as images for testing, while the synthetic dataset consists of labeled images for training. We consider the lightbox dataset and sunlamp dataset as target dataset, while the synthetic dataset serves as the source dataset.

#### 4.1.2. Soyuz Dataset

The URSO dataset is synthesized using Unreal Engine-4 [15]. The dataset describes various poses of two satellites (Dragon, Soyuz) during their orbit around the Earth. Dragon is a cylindrical spacecraft, which is not applicable to the scope of this study. The dataset for Soyuz is divided into two parts, Soyuz_easy and Soyuz_hard. Soyuz exhibits solar panels with distinct coplanar features. In Soyuz_easy, the quantities for the training dataset, validation dataset, and testing dataset are 4000, 500, and 500, respectively. For Soyuz_hard, the corresponding quantities are 3909 for the training dataset, 489 for the validation dataset, and 490 for the testing dataset.

### 4.2. Evaluation on the SPEC2021 Dataset

**Figure 4:**
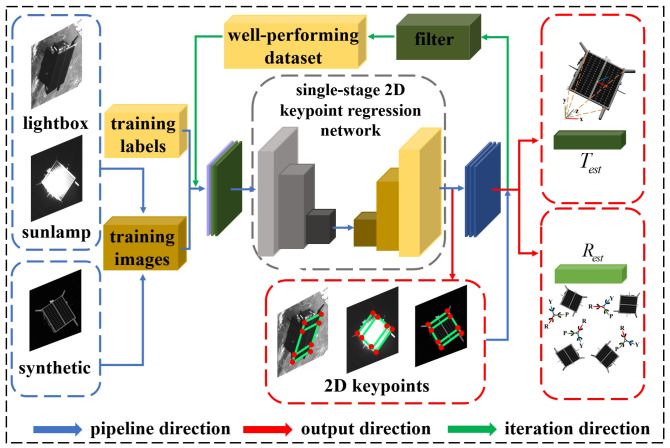
Illustrating the single-stage 2D keypoint regression network for the SPEC2021 dataset with semi-supervised domain adaptation.

SPEC2021 particularly emphasizes domain gaps where the source and target data distributions differ, while the tasks remain the same. We adopt a semi-supervised learning strategy [37,38,39]. Specifically, we randomly select a portion of the target dataset and annotate it in the format of coco [34]. Moreover, we perform image augmentation operations on the annotated dataset, such as rotation, translation, scaling, occlusion, and combinations of these operations. The aim is to enhance the robustness and stability of the network model. After applying image augmentation, both the augmented target dataset and the source dataset are fed into the single-stage 2D keypoint regression network for training. We use the trained model to test the target dataset, filter out the well-performing dataset, incorporate the results into the training dataset, and input them into the network for further training, as depicted in Figure 4. This process is repeated iteratively, ultimately selecting a model with better weights.

**Figure 5:**
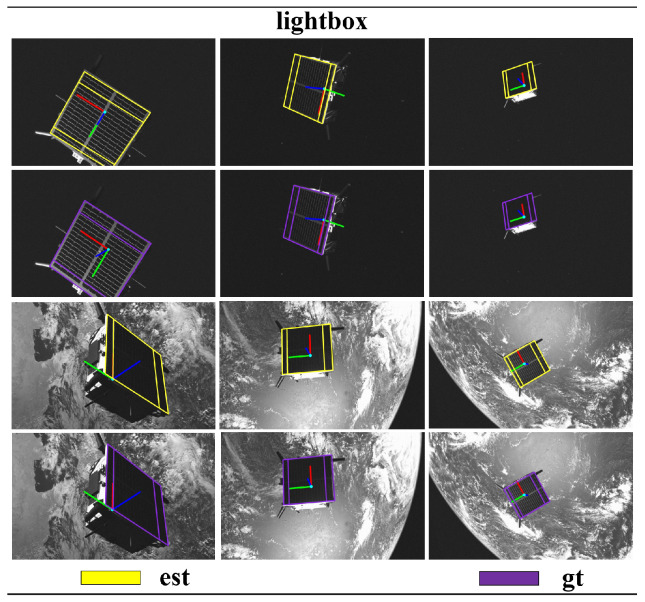
In the SPEC2021 lightbox dataset, a comparison is conducted between the pose obtained using our proposed research method (est) and the pose ground truth (gt).

**Figure 6:**
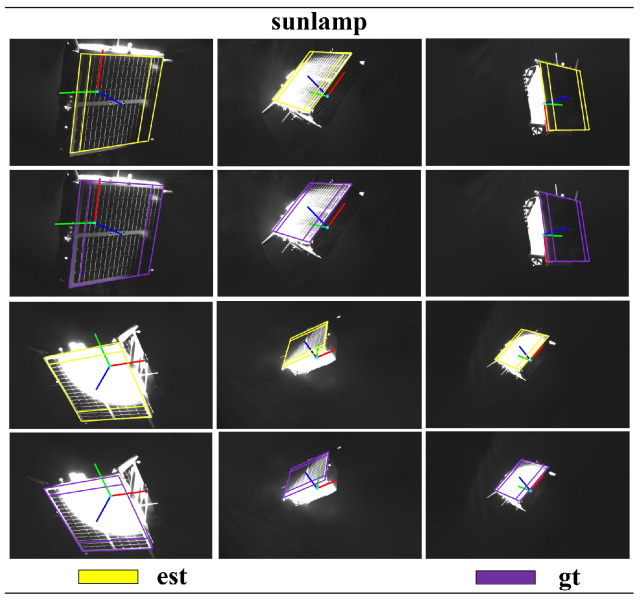
In the SPEC2021 sunlamp dataset, a comparison is conducted between the pose obtained using our proposed research method (est) and the pose ground truth (gt).

**Figure 7:**
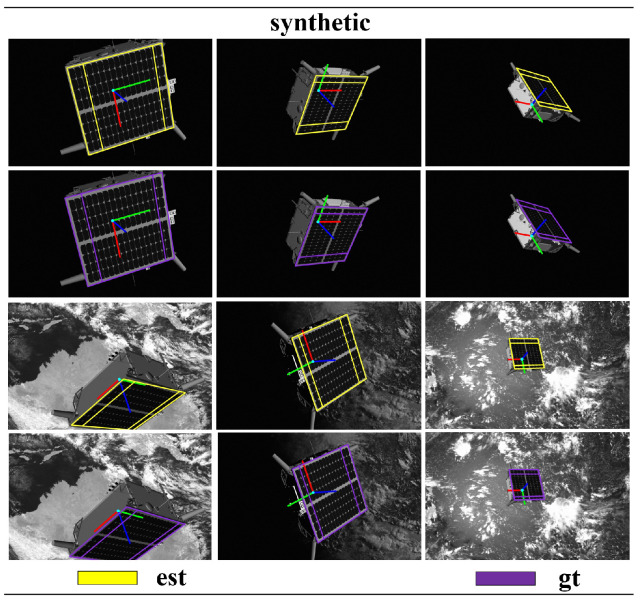
In the SPEC2021 synthetic dataset, a comparison is conducted between the pose obtained using our proposed research method (est) and the pose ground truth (gt).

The test results of the target dataset are compared with the top five results in the SPEC2021 post-mortem, as shown in Table 1 and Table 2. Although our pose score is not the highest, the performance is relatively commendable. Other methods show significant differences in pose scores between the lightbox dataset and sunlamp dataset tests. In contrast, our method exhibits smaller differences in the tests on the lightbox dataset and sunlamp dataset, indicating that our approach is more robust and generalizes well. Additionally, we compared our method with two-stage method [40,41] on synthetic images. Our method did not achieve higher pose scores compared to the two-stage method, for two main reasons. Firstly, the two-stage method utilizes two networks: the first-stage sub-network detects the target in the image and provides region-of-interest proposals, while the second-stage sub-network regresses keypoints of the target within the regions of interest [17,42]. In contrast, our proposed single-stage network conducts both target detection and keypoint regression across the entire image, which poses certain challenges. Secondly, the two-stage method globally models the 3D skeleton of the Tango spacecraft, whereas our method locally models the 3D skeleton of the Tango spacecraft focusing on local coplanar components (such as solar panels), allowing the two-stage method to capture all features of the target. In summary, these are the reasons why the performance of the two-stage method surpasses that of our proposed method. However, the network of the two-stage method comprises two sub-networks, and global 3D modeling presents greater complexity than local coplanar modeling. This paper aims to reduce the complexity of the spacecraft pose estimation network while achieving accurate pose computation using simple 3D geometric models as much as possible. Compared to SPN [10] and KRN [21], our proposed single-stage spacecraft pose estimation research method demonstrates more advanced capabilities, as shown in Table 3. We reproject the estimated poses onto the images and compare them with the ground-truth poses, as illustrated in Figure 5, Figure 6 and Figure 7.

### 4.3. Evaluation on the URSO Soyuz Dataset

**Figure 8:**
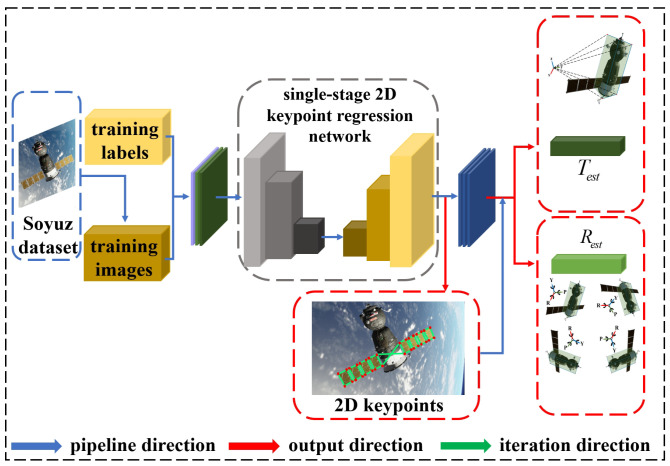
Illustrating the single-stage 2D keypoint regression network for the Soyuz dataset.

**Figure 9:**
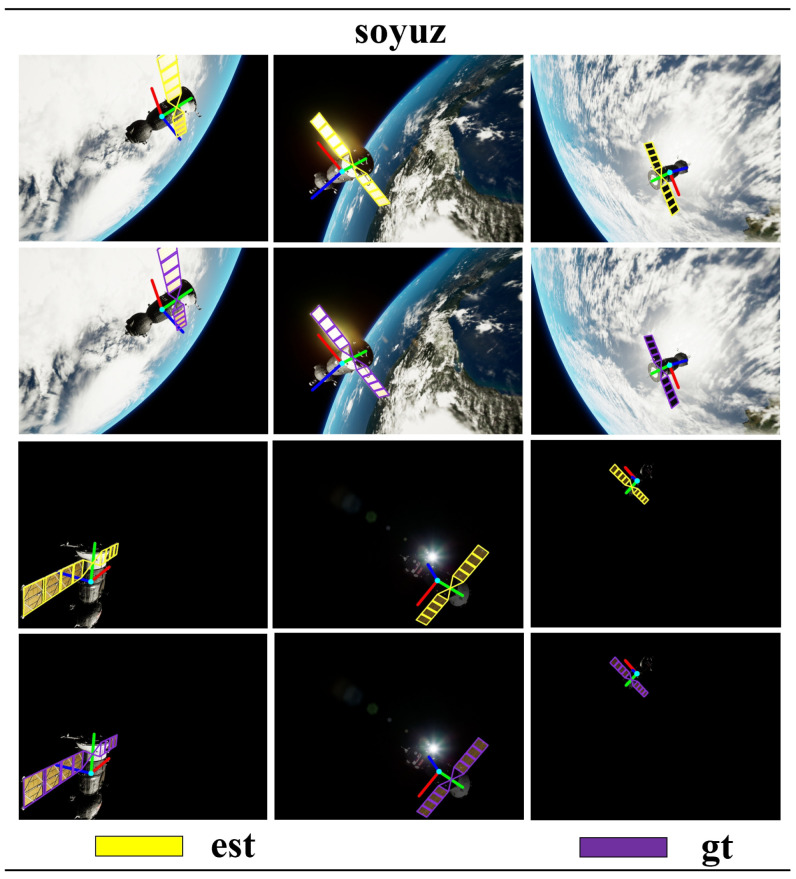
In the URSO Soyuz dataset, a comparison was conducted between the pose obtained using our proposed research method (est) and the pose ground truth (gt).

We chose the Soyuz spacecraft in the URSO dataset as our research subject. We selected the solar panels of Soyuz to construct a homographic geometric constraint equation for solving the Soyuz spacecraft pose, as depicted in Figure 8. Our test results are presented in Table 4, showing that, compared to UrsoNet [15], our proposed research method demonstrated improved accuracy in both position and orientation, particularly in terms of orientation precision. We conducted pose estimation on the Soyuz dataset for training, testing, and validation, and the results were evaluated based on the Average Distance of Corresponding Points ($\omega_{ADD}$). For Soyuz_easy, the obtained $\omega_{ADD}$ was above 90%, while for Soyuz_hard, the calculated $\omega_{ADD}$ was above 80%, as shown in Table 5. Additionally, we reprojected the estimated poses onto images and compared them with the ground-truth poses, as illustrated in Figure 9.

### 4.4. Backbone and Runtime Performance

**Figure 10:**
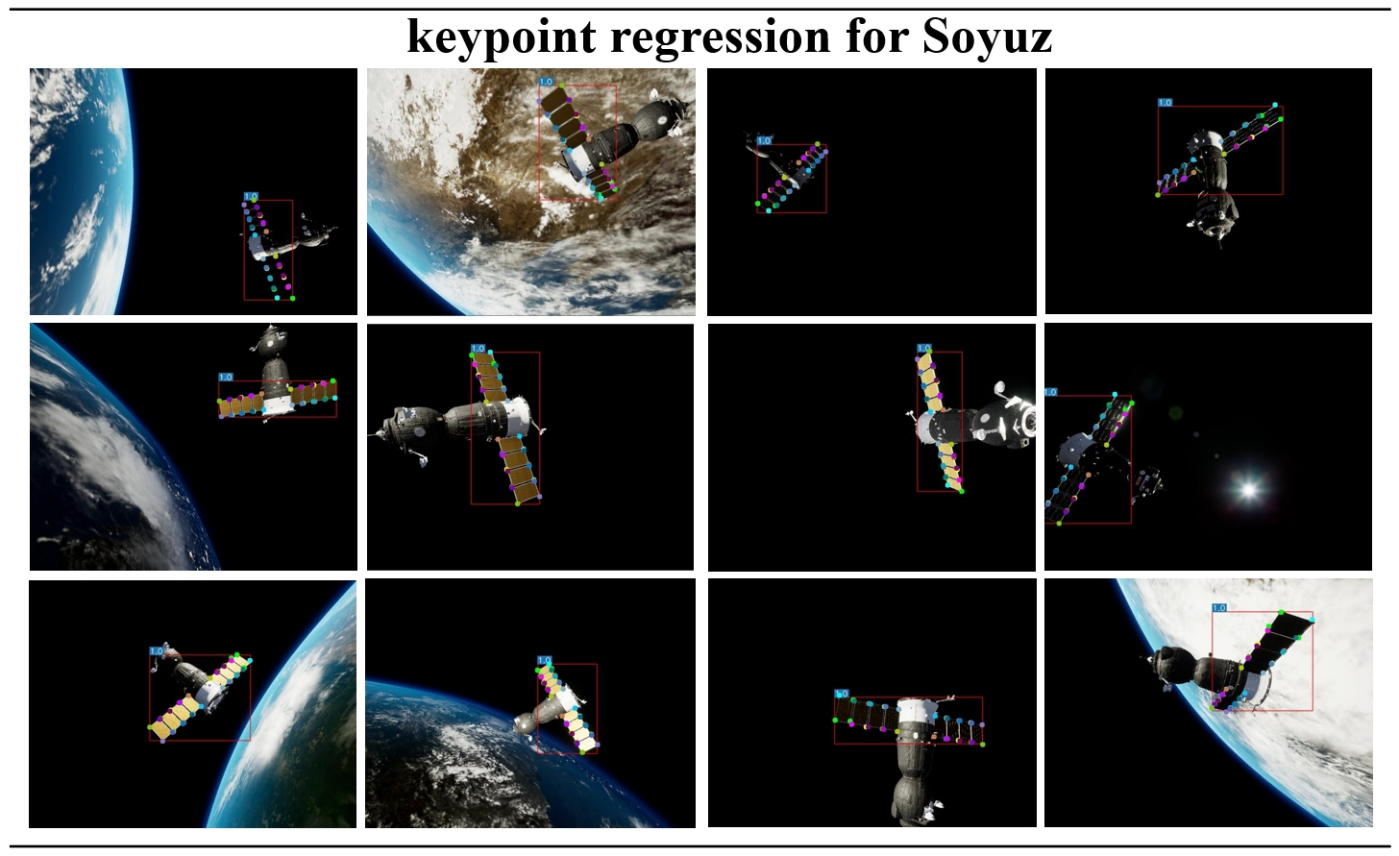
Illustrating the keypoint regression for the Soyuz spacecraft solar panels in the image through a single-stage spacecraft pose network.

We performed a performance analysis on the single-stage spacecraft pose network using Soyuz as the research subject. The results of pose estimation accuracy obtained by changing the input image size are presented in Table 6. The findings indicate that as the input image size increased, the position accuracy improved correspondingly, but the orientation accuracy decreased. The results of keypoint regression for spacecraft using the single-stage spacecraft pose network are shown in Figure 10. We conducted experiments on a PC equipped with NVIDIA GeForce RTX 3080 Ti and compared our method with the two-stage approach. Almost invariably, the two-stage method exhibited larger network Params, GFLOPs, and single-frame runtime compared to our single-stage network. This demonstrates that our approach achieved higher operational efficiency and lower computational complexity, as indicated in Table 7.

### 4.5. Evaluation of Datasets and Results

Two different datasets (SPEC2021 Tango and URSO Soyuz) were used to validate the proposed method in this paper. To evaluate the effect of different datasets on pose estimation performance, we conducted comparative experiments on these two datasets, as depicted in Table 8. The experiments were designed to evaluate the influence of different datasets on pose estimation performance based on image type and the number of keypoints. The datasets for lightbox and sunlamp were introduced in Section 4.1, while the datasets for SPEC2021 and URSO Soyuz were synthetically generated using simulator software [14,15]. URSO Soyuz has two solar panels, one on each side of the Soyuz main body, which allows for eight keypoints based on this structure. Each solar panel consists of four subpanels, resulting in a total of 32 keypoints based on this structure. Different datasets contain images with different styles, which can impact the results of pose estimation. Additionally, in different datasets, richer information about keypoints of the target is more conducive to improving the accuracy of pose estimation.

## 5. Conclusions

This paper proposes a single-stage-network-based method for spacecraft homography pose estimation. Compared to commonly used spacecraft pose estimation networks, our proposed approach accomplishes both detection and classification tasks within a single network. In contrast to two-stage-network-based spacecraft pose estimation methods, the method presented in this paper employs a single-stage network with lower computational complexity and higher operational efficiency. Unlike methods that construct high-precision global models of spacecraft, our proposed approach focuses on estimating the pose of coplanar components of spacecraft, such as solar panels or antennas, which reduces the difficulty of constructing models for local coplanar components of spacecraft. Additionally, our method remains effective in scenarios with domain gaps or challenges.

## Figures and Tables

**Table 1:** Our approach compared to other results in the SPEC2021 post-mortem for lightbox.

Methods	Lightbox		
	$S_{q}\;\left[\mathrm{rad}\right]$	$S_{t}$	S
mystery_team [39]	0.030	0.009	0.039
haoranhuang_njust	0.051	0.014	0.065
TangoUnchained	0.052	0.016	0.068
prow	0.094	0.020	0.114
vpu_post_mortem [38]	0.093	0.027	0.120
ours	0.050	0.018	0.068

**Table 2:** Our approach compared to other results in the SPEC2021 post-mortem for sunlamp.

Methods	Sunlamp		
	$S_{q}\;\left[\mathrm{rad}\right]$	$S_{t}$	S
haoranhuang_njust	0.047	0.011	0.058
lava1302 [37]	0.047	0.012	0.059
mystery_team [39]	0.046	0.013	0.059
prow	0.084	0.013	0.097
vpu_post_mortem [38]	0.087	0.023	0.110
ours	0.048	0.014	0.062

**Table 3:** Synthetic-only performances of the methods with tested on synthetic validation images.

Methods	Δt[m]	$S_{q}\;{[}^{\circ }]$	*S*
SPN [10]	0.16	7.77	0.16
KRN [21]	0.14	3.69	0.09
HigherHRnet+EPnP [39]	0.05	1.51	0.04
ours	0.10	2.34	0.06

**Table 4:** Our approach compared to UrsoNet results in the Soyuz_easy dataset and Soyuz_hard dataset.

Methods	Dataset	Δt[m]	$S_{q}\;{[}^{\circ }]$
UrsoNet [15]	Soyuz_easy	–	–
	Soyuz_hard	0.80	7.70
ours	Soyuz_easy	0.72	3.44
	Soyuz_hard	0.73	5.02

**Table 5:** Pose estimation accuracy evaluation (ADD) on the Soyuz dataset for training, testing, and validation.

Soyuz_easy		Soyuz_hard	
**Set**	**ADD**	**Set**	**ADD**
training	96.8	training	90.2
testing	94.4	testing	81.0
validation	92.0	validation	82.2

**Table 6:** Performance comparison at different image resolutions. We utilized the Soyuz dataset for our analysis.

Resolution	Δt[m]	$S_{q}\;{[}^{\circ }]$
640×640	0.73	5.02
960×960	0.65	5.35

**Table 7:** Comparison of methods on the Soyuz dataset regarding model size, computational complexity, and runtime.

Network	Methods	Params/M	GFLOPs/G	Times/ms
Single-stage	yolo-w6-pose	80.5	102.3	47.7
	yolo-tiny-pose	9.8	20.4	43.9
Two-stage	yolov7, hrnet_32	65.0	110.3	127.4
	yolov7, hrnet_48	100.1	136.1	150.4

**Table 8:** Evaluating the effect on the pose estimation performance for different datasets.

Dataset	Image Type	Keypoints	ADD
SPEC2021 Tango	lightbox	8	71.7
	sunlamp	8	80.8
	synthetic	8	73.8
URSO Soyuz	synthetic	8	81.5
	synthetic	32	87.4

## Data Availability

The data presented in this study are openly available in https://kelvins.esa.int/pose-estimation-2021/data/ and https://github.com/pedropro/UrsoNet?tab=readme-ov-file.
